# Explainable machine learning with wavelet‐enhanced multi‐scale texture analysis of diabetes‐related heel pad ultrasound texture differences

**DOI:** 10.1002/acm2.70888

**Published:** 2026-11-05

**Authors:** Xinlei Ji, Yiming Li, Jiachen Guo, Yitian Zhang, Liang Zhou, Tao Wu, Lei Yin

**Affiliations:** ^1^ School of Health Science and Engineering University of Shanghai for Science and Technology Shanghai China; ^2^ Centre for Collaborative Research Shanghai University of Medicine and Health Sciences Shanghai China; ^3^ Department of Ultrasound Huashan Hospital Fudan University Shanghai China; ^4^ Suzhou Xiacheng Intelligent Technology Co. Ltd. Suzhou China; ^5^ School of Electronic and Electrical Engineering Shanghai University of Engineering Science Shanghai China; ^6^ JiangWan Hospital of Hongkou district Shanghai University of Medicine and Health Sciences Shanghai China; ^7^ Shanghai University of Traditional Chinese Medicine Shanghai China; ^8^ Faculty of Intelligence Technology Shanghai Institute of Technology Shanghai China

**Keywords:** diabetes mellitus, explainable artificial intelligence, heel pad ultrasonography, machine learning, ultrasound texture analysis

## Abstract

**Background:**

Diabetes is associated with structural and functional differences in plantar soft tissues, which may be reflected in ultrasound texture patterns. However, existing ultrasound texture studies are limited by single‐scale feature extraction and limited feature‐level interpretability.

**Purpose:**

To develop an explainable machine learning framework incorporating a wavelet‐enhanced multi‐scale texture feature extraction module (WEMS‐TFE), and to investigate whether heel pad ultrasound texture features differ between patients with diabetes without active foot ulcers and healthy controls.

**Methods:**

Heel pad ultrasonography was performed in 76 patients with diabetes (without active foot ulcers) and 47 healthy controls. Ultrasound images of the heel pad were analyzed at the microchamber and macrochamber levels. The WEMS‐TFE module was proposed to extract multi‐scale, multi‐directional texture features via two‐dimensional discrete wavelet decomposition combined with the gray‐level co‐occurrence matrix (GLCM). Features with significant between‐group differences were selected and evaluated across seven machine learning classifiers using repeated cross‐validation with participant‐disjoint data partitions. SHapley Additive exPlanations (SHAP) analysis was used to interpret feature contributions in a representative XGBoost model.

**Results:**

The WEMS‐TFE scheme achieved the highest cross‐model composite performance and robustness among the five evaluated feature extraction schemes. Cross‐classifier importance analysis identified 6 features that recurred across multiple classifiers. Consistently, most of these also ranked among the important features in the XGBoost SHAP analysis, and two representative features showed clear value‐dependent contribution patterns. The identified features therefore reflected group‐associated image‐texture differences, although their associations may be affected by residual demographic confounding.

**Conclusions:**

The proposed framework, with the WEMS‐TFE module as its core component, enables quantitative characterization of group‐associated differences in heel pad ultrasound texture features. The findings provide imaging‐based evidence of group‐level texture differences in the heel pad and support further investigation of plantar tissue characteristics in diabetes, while acknowledging that these differences may be affected by residual demographic confounding and do not establish diabetes‐specific structural changes or causal tissue mechanisms.

## 1 INTRODUCTION

Diabetic foot (DF) is a major complication of diabetes, with ulceration and tissue destruction representing important clinical manifestations.^1^ Because plantar tissue characteristics may differ between individuals with diabetes and healthy individuals, quantitative characterization of plantar tissue using ultrasound may provide complementary information for understanding diabetes‐associated tissue differences.

The core pathogenesis of DF is driven by peripheral neuropathy and peripheral arterial disease (PAD) as underlying factors, compounded by infection and biomechanical damage.^2^ Diabetes may also affect the composition and mechanical organization of plantar soft tissues through chronic metabolic and vascular changes.^3^ These observations indicate that plantar soft tissues may exhibit measurable differences in patients with diabetes, but such differences are not fully captured by conventional morphological measurements.

Multiple studies using dynamometry, elastography, and ultrasound have identified structural or functional differences in plantar tissues in patients with diabetes.^4^, ^5^, ^6^ Ultrasound offers a non‐invasive, real‐time, and reproducible means of assessing plantar soft‐tissue morphology and has therefore been widely used for plantar tissue characterization.^7^ However, conventional measurements such as tissue thickness and visual morphological assessment may not fully capture the diffuse spatial heterogeneity of plantar tissues. Because diabetes‐related soft‐tissue differences may be distributed across the tissue rather than confined to discrete focal lesions, they may not be adequately characterized by gross morphological descriptors alone.

Ultrasound texture analysis offers a promising route toward automated, quantitative assessment of plantar soft tissue beyond conventional morphological measurements. By characterizing gray‐level distributions and spatial relationships, texture features can quantify image‐level heterogeneity that may be difficult to capture through visual assessment alone. Duan et al. applied the gray‐level co‐occurrence matrix (GLCM) to extract texture features and found that the correlation, energy, and entropy of the macrochamber in diabetic heel pads differed significantly from those of healthy controls.^8^ Puri et al. used wavelet‐based texture energy features that correlated strongly with plantar stiffness and effectively distinguished healthy, diabetic, and ulcerated plantar tissue, reflecting subcutaneous microstructure.^9^ However, these studies relied on individual texture extraction strategies and limited‐scale representations, with limited cross‐model validation and feature‐level interpretation. Given that plantar ultrasound textures contain information across multiple spatial scales and orientations, a more comprehensive feature representation and systematic evaluation may provide a more complete characterization of diabetes‐associated texture differences.

To address these limitations, we developed an interpretable machine learning framework for heel pad ultrasound images. Heel pad ultrasound images were collected from patients with diabetes and healthy controls; image preprocessing and segmentation of the microchamber and macrochamber layers were performed using H‐SAM.^10^ GLCM provides statistical descriptions of spatial relationships among pixel intensities and has been widely applied to ultrasound texture analysis,^11^, ^12^, ^13^, ^14^ whereas two‐dimensional discrete wavelet transform (2D‐DWT) decomposes ultrasound images into sub‐bands at multiple scales and orientations, providing multi‐scale texture representations.^15^ These complementary spatial‐statistical and multi‐scale representations motivated the proposed Wavelet‐Enhanced Multi‐scale Texture Feature Extraction (WEMS‐TFE) module, which integrates multi‐scale decomposition via 2D‐DWT with multi‐directional GLCM texture feature extraction.^16^ The resulting feature representation was subjected to statistical feature selection and evaluated across seven machine learning classifiers representing different modeling mechanisms, allowing the consistency of the captured discriminative information to be examined across models. Finally, feature importance analysis and SHapley Additive exPlanations (SHAP) were used to characterize feature contributions to model predictions.

Despite the increasing use of machine learning in medical image analysis, model interpretability remains important for understanding which image‐derived features contribute to predictions.^17^, ^18^, ^19^ For texture‐based models, this is particularly relevant because the relationship between predefined image features and model outputs may not be readily apparent. SHAP, a widely used post‐hoc explanation method grounded in cooperative game theory, quantifies the contribution of individual features to model predictions and can reveal value‐dependent contribution patterns.^20^, ^21^, ^22^, ^23^, ^24^ However, feature‐level interpretation of plantar ultrasound texture models remains limited, particularly regarding the consistency of feature importance across different classifiers.

The main contributions of this study, which address these gaps, are as follows:

(1) We establish a systematic analysis framework for characterizing heel pad ultrasound texture differences, integrating multi‐scale feature extraction, statistical feature selection, cross‐model validation, and feature‐level interpretation.
(2) We propose the WEMS‐TFE module, which integrates the multi‐scale analysis capability of 2D‐DWT with the statistical texture characterization of GLCM to capture texture information across multiple spatial scales and orientations.
(3) We integrate multi‐scale texture analysis with cross‐model feature importance analysis and SHAP‐based interpretation for plantar ultrasound imaging, enabling the identification of consistently important texture features and the characterization of their contributions to model predictions.

Therefore, this study aimed to investigate whether multi‐scale texture features extracted from heel pad ultrasound images could discriminate between patients with diabetes without active foot ulcers and healthy controls. We further aimed to evaluate the contribution of the WEMS‐TFE representation through predefined cross‐model performance and robustness measures, identify features that showed consistent importance across different classifiers, and use SHAP analysis to characterize model‐specific feature contributions.

## 2 MATERIALS AND METHODS

This study developed an explainable machine learning framework for heel pad ultrasound images (Figure 1) designed to characterize group‐associated differences in heel pad ultrasound texture between patients with diabetes and healthy controls. The framework consists of five steps: (1) data preprocessing and segmentation, in which the heel pad microchambers and macrochambers were delineated; (2) multi‐scale texture feature extraction, in which the proposed WEMS‐TFE module extracted texture features from both regions using 2D‐DWT and GLCM; (3) statistical difference screening and feature selection, in which between‐group differences and feature redundancy were evaluated; (4) machine learning classification, in which seven complementary models were used to evaluate the discriminative consistency and robustness of the selected feature representation; and (5) interpretability analysis, in which feature importance and SHAP analysis were used to characterize the contributions and value‐dependent patterns of key features.

**FIGURE 1:**
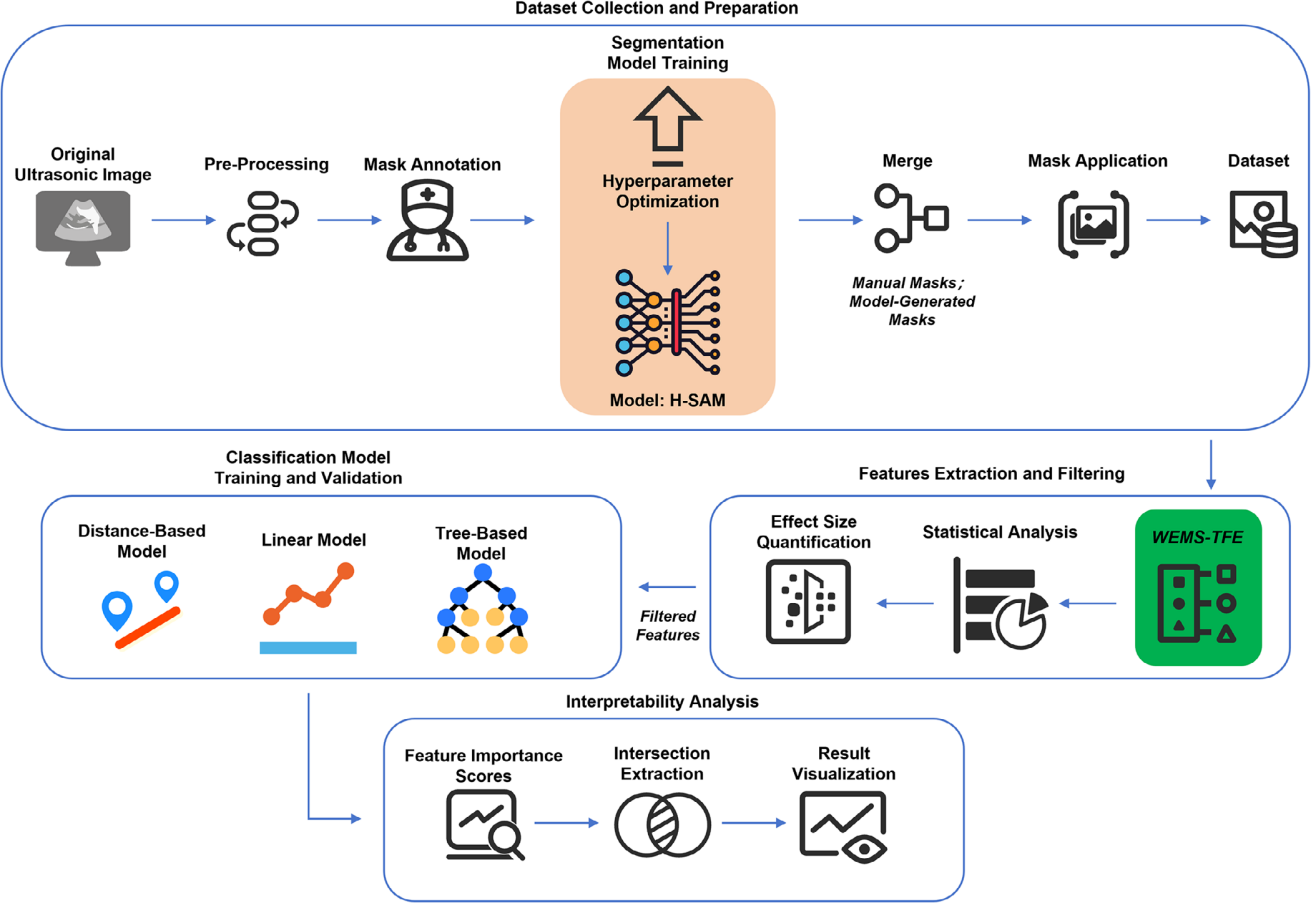
Overall workflow of the proposed framework for heel pad ultrasound analysis.

### 2.1 Participants and ultrasound image acquisition

This study was conducted in accordance with the Declaration of Helsinki. Imaging data were collected with all data strictly anonymized. This prospective study was approved by the relevant institutional ethics review board. All participants provided informed consent.

From August 2022 to December 2024, 76 patients with diabetes and 47 healthy controls from the community were enrolled. None of the patients with diabetes had active foot ulcers or wounds at the time of ultrasound examination. Diabetes type was not systematically recorded. Peripheral neuropathy was assessed by experienced clinicians using the Neuropathy Disability Score (NDS), with a score ≥ 3 considered indicative of diabetic peripheral neuropathy. NDS assessments were unavailable for 11 patients with diabetes. PAD status was not collected.

A total of 289 two‐dimensional plantar ultrasound images were obtained from the 123 participants, including 156 images from the diabetes group and 133 from the healthy control group. Multiple images were collected from some participants.

All ultrasound images were acquired using an Aplio i900 ultrasound system (Canon Medical Systems, Japan) equipped with a broadband linear‐array transducer (frequency range, 5–18 MHz) optimized for musculoskeletal ultrasonography. To minimize the potential confounding effects of joint positioning and mechanical loading on plantar soft tissue characteristics, participants were examined in a lateral recumbent position with the knee flexed and the ankle maintained in a relaxed, unloaded state. Ultrasound examinations were independently performed by two certified sonographers, each with more than 10 years of clinical experience and specialized training in musculoskeletal ultrasonography. Under real‐time ultrasound guidance, the calcaneal tuberosity was identified to ensure consistent anatomical localization of the heel region across all participants. Ultrasound images were acquired from the right heel region under unloaded conditions to preserve the native morphological characteristics of the plantar soft tissues.

Although images from the same participant shared similar anatomical structures and overall texture patterns, they were not identical because of slight variations in probe position, scanning angle, and imaging plane. To account for the non‐independence of images from the same participant, all images from each participant were assigned exclusively to the same data subset, thereby preventing data leakage between the training, validation, and test sets.

### 2.2 ROI extraction and inter‐observer agreement evaluation

Since the original ultrasound images contained substantial non‐diagnostic information, including blank areas, system interface elements, measurement markers, acoustic artifacts, and physician‐added annotations, Region of Interest (ROI) extraction was performed before model training. The ROI boundaries were manually delineated using a dedicated image processing tool under the guidance of ultrasound physicians to include the complete plantar soft tissue region of the heel while excluding irrelevant areas. All extracted ROIs were reviewed to ensure consistency and preservation of anatomical structures. The reproducibility of manual ROI extraction was evaluated by three independent observers (two trained research investigators and one ultrasound physician) using the intersection‐over‐union (IoU) metric.

### 2.3 Anatomically Guided Heel Pad Segmentation and Dataset Preparation

The heel fat pad exhibits a well‐defined bilaminar architecture comprising a microchamber and a macrochamber. The macrochamber is a foundational structure formed during embryonic development, in which relatively coarse fibrous septa delineate large chambers, whereas the microchamber is a finer structure that matures during adulthood, in which delicate septa further subdivide the tissue into smaller compartments rich in elastic fibers. These two layers differ markedly in adipocyte size, fibrous composition, and mechanical properties: the microchamber, which is stiffer, primarily provides structural support, whereas the macrochamber, which has greater deformability, serves as the principal shock‐absorbing and load‐cushioning element. Together, they form the integrated mechanical cushioning system of the heel.^25^, ^26^, ^27^

The microchamber and macrochamber differ substantially in structural and mechanical properties and are partially distinguishable in ultrasound images (Figure 2). These distinctions enable clinicians to approximate the segmentation boundary and spatial extent of each layer through visual inspection guided by clinical experience. High‐quality segmentation mask datasets can therefore be constructed using standard image annotation tools. However, manual annotation is labor‐intensive and time‐consuming, rendering it impractical in clinical practice. A more efficient approach is consequently needed.

**FIGURE 2:**
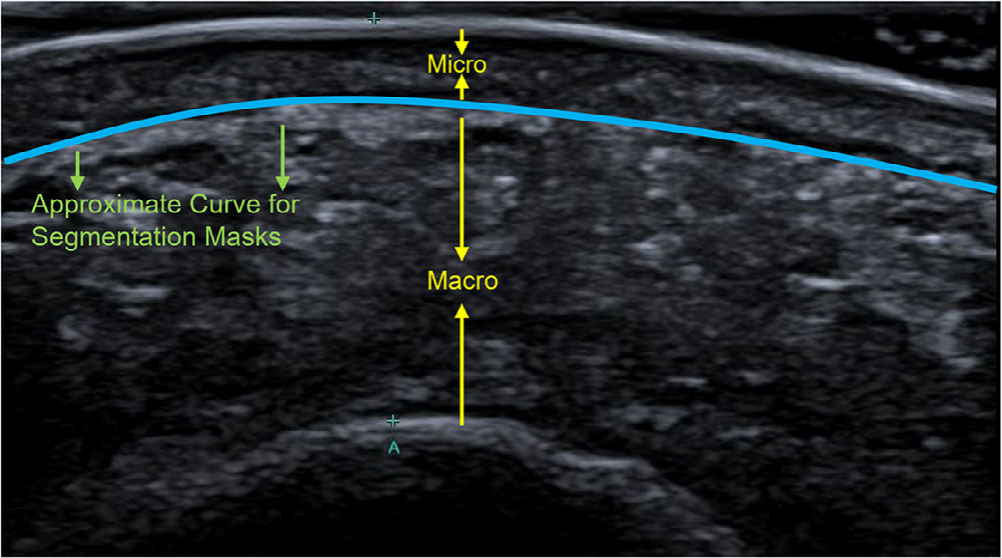
Annotated ultrasound images illustrating the boundaries and spatial extents of the microchamber and macrochamber in the heel pad.

This study employed H‐SAM, an advanced medical image segmentation model built upon the pretrained SAM framework. H‐SAM incorporates low‐rank adaptation (LoRA) to enable few‐shot learning and introduces an innovative coarse‐to‐fine two‐stage hierarchical decoding module, achieving superior performance across multiple medical imaging benchmarks. In this study, 46 masks were manually annotated by experienced physicians and derived from 23 images of 12 patients with diabetes and 23 images of 12 healthy controls. Eight images from four participants (two patients with diabetes and two healthy controls) were held out as an independent test set, with these participants excluded from all training and validation procedures. The remaining 38 from the other participants were partitioned at the participant level for five‐fold cross‐validation (∼30 training and ∼8 validation images per fold) to prevent data leakage. Within each fold, hyperparameters were optimized by grid search with early stopping to mitigate overfitting; the optimal configuration and its early‐stopping epoch were recorded. To maximize data utilization, the model was then retrained on all 38 images for the recorded number of epochs, without a separate validation split. Finally, the trained H‐SAM model was applied to the remaining unannotated images to generate segmentation masks, which were merged with the manual masks and overlaid on the original images to segment both layers.

### 2.4 GLCM‐based texture feature extraction

This study selected 0°, 45°, 90°, and 135° as the core orientations for the GLCM, based on the fundamental spatial relationships of the eight‐neighbor pixel configuration; these four directions provide complete coverage of the horizontal, vertical, and diagonal principal texture structural features with minimal redundancy. By extracting texture features along multiple orientations and constructing a joint feature vector, this approach captures the directional dependence of texture across the principal orientations, providing a computationally efficient representation of directional texture characteristics.

This study adopted the 14 GLCM‐based features proposed by Haralick et al.^28^ for image texture characterization, which encompass multi‐dimensional characterization metrics. Built upon the GLCMs constructed at the four orientations described above, these features fully capture the spatial dependence of pixel gray‐level values and the underlying textural statistical regularities. The specific mathematical definitions and computational formulae for each texture feature are detailed in Appendix A.

### 2.5 Wavelet‐enhanced multi‐scale texture feature extraction

#### 2.5.1 Two‐level wavelet decomposition

The 2D‐DWT was implemented using the wavedec2 function from the PyWavelets library (version 1.7.0),^29^ with the Haar wavelet selected as the mother wavelet because of its low computational complexity, compact support, and ability to characterize local intensity transitions and edge‐related structures.

In discrete implementation, the wavelet transform is realized through a set of orthogonal filter banks. For the Haar wavelet, the corresponding low‐pass and high‐pass filters are defined as:

(1) $$h\left(n\right)=\left\{\begin{array}{cc} \frac{1}{\sqrt{2}}, & n=0,1\\ 0, & \mathrm{elsewhere} \end{array}\right.$$

(2) $$g\left(n\right)=\left\{\begin{array}{cc} \frac{1}{\sqrt{2}}, & n=0\\ -\frac{1}{\sqrt{2}}, & n=1\\ 0, & \mathrm{elsewhere} \end{array}\right.$$

where h(n) denotes the low‐pass filter, g(n) denotes the high‐pass filter, and n takes integer values.

Based on these filters, the 2D‐DWT decomposes the input image into four sub‐bands, as expressed in Equations (3)–(6).

(3) $$LL=↓2\left(↓2\left(I*h^{T}\right)*h\right)$$

(4) $$LH=↓2\left(↓2\left(I*h^{T}\right)*g\right)$$

(5) $$HL=↓2\left(↓2\left(I*g^{T}\right)*h\right)$$

(6) $$HH=↓2\left(↓2\left(I*g^{T}\right)*g\right)$$

Here, I represents the input image, h and g represent the one‐dimensional low‐pass and high‐pass filters, respectively, and ∗ denotes one‐dimensional convolution. T denotes the transpose of the filter, used for row convolution, and ↓2 denotes downsampling (stride = 2). Each sub‐band has half the height and width of its input image.

The four sub‐bands comprise the low‐frequency approximation component (LL), horizontal detail component (HL), vertical detail component (LH), and diagonal detail component (HH).

As illustrated in Figure 3, the first‐level decomposition generated one approximation sub‐band (LL1) and three detail sub‐bands (LH1, HL1, and HH1). The approximation sub‐band LL1 was then further decomposed to obtain LL2, LH2, HL2, and HH2. The detail sub‐bands from both decomposition levels were retained for texture analysis, whereas the approximation sub‐bands were excluded. The first‐ and second‐level detail sub‐bands represent relatively fine‐ and coarse‐scale texture information, respectively, providing a multi‐scale representation of the ROI.

**FIGURE 3:**
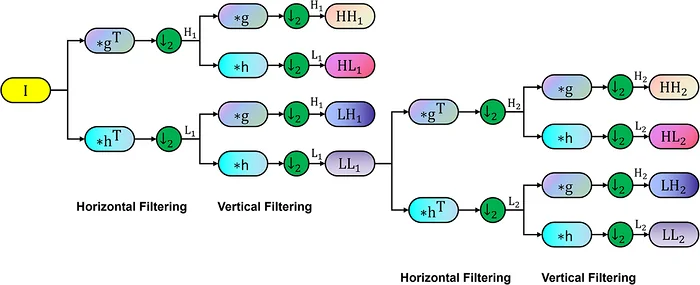
Two‐level decomposition for the 2D‐DWT.

#### 2.5.2 Directional GLCM feature extraction and integration

Before GLCM calculation, each detail sub‐band was independently normalized using min‐max scaling to the range of 0–255 and converted to an 8‐bit unsigned integer image (uint8). The normalization was performed using the minimum and maximum coefficient values of each sub‐band, with all values set to zero when the maximum and minimum values were equal. The normalized values were converted to integers by truncation, resulting in a nominal gray‐level range of 0–255 (256 possible gray levels).

For each detail sub‐band, 14 Haralick texture features were calculated using the haralick function from the mahotas.features.texture module in Python (3.11.8) (mahotas version 1.4.18), with the pixel‐pair distance set to 1 and compute_14th_feature = True. Four GLCM directions (0°, 45°, 90°, and 135°) were considered. To account for the directional characteristics of the wavelet detail sub‐bands while reducing redundant directional combinations, a sub‐band–direction selection strategy was adopted. According to the implemented feature‐selection procedure, the 0° features were retained from HL, the 45° and 135° features from LH, and the 90° features from HH. This selection was applied consistently at both decomposition levels. The ignore_zeros = True setting was used to exclude zero‐valued pixels from the corresponding GLCM calculations.

Each decomposition level contributed 56 features, comprising 14 features from HL, 28 from LH, and 14 from HH. Across the two decomposition levels, this yielded 112 features for each ROI. The procedure was performed independently for the cropped microchamber and macrochamber ROIs. The resulting 112‐dimensional vectors from the two ROIs were concatenated to form a 224‐dimensional image‐derived feature vector for each ultrasound image, as illustrated in Figure 4.

**FIGURE 4:**
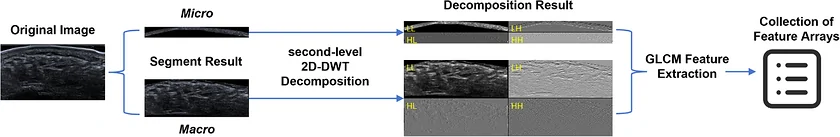
WEMS‐TFE process: Two‐level 2D‐DWT decomposition, GLCM feature extraction on sub‐bands from both levels, and collection of feature vectors.

### 2.6 Sensitivity of texture features to segmentation error

To assess the robustness of the WEMS‐TFE features to segmentation uncertainty, we simulated boundary under‐ and over‐segmentation by applying morphological erosion (−2 and −5 px) and dilation (+2 and +5 px) to the reference masks. The same feature set was extracted from each perturbed mask using the identical pipeline.

Feature stability was quantified by (1) the intraclass correlation coefficient (ICC) between perturbed and reference values, computed under a two‐way random‐effects model with absolute agreement [ICC(2,1)], and (2) the relative change (%), defined per participant as

$$\frac{\left|x_{\mathrm{perturbed}}-x_{\mathrm{reference}}\right|}{\left|x_{\mathrm{reference}}\right|}\times 100$$

ICC values were interpreted as poor (< 0.50), moderate (0.50–0.75), good (0.75–0.90), and excellent (≥ 0.90).^30^ For each perturbation scheme, we reported the median ICC, ICC interquartile range (IQR), the proportion of features with ICC ≥ 0.90, and the median and IQR of the relative change. Features with zero variance in either condition were excluded from ICC computation. All analyses were performed using NumPy 1.26.4, pandas 2.2.3, and SciPy 1.14.1 in Python 3.11.8.

### 2.7 Statistically‐based feature filtering

After extracting texture features from the microchambers and macrochambers of all ultrasound images, not all of the initially extracted multi‐dimensional features contribute effectively to class discrimination. Therefore, conducting differential analysis through statistical methods to filter out features without significant differences not only helps retain discriminative information but also effectively removes redundant and noisy features.

To evaluate feature differences between patients with diabetes and healthy controls, with patients with diabetes defined as the experimental group and healthy controls as the control group, 14 Haralick features were analyzed across four directions (0°, 45°, 90°, and 135°) and two heel pad regions (microchambers and macrochambers). For a given image representation, these features yielded 112 features (14 Haralick features × 4 directions × 2 regions). For representations incorporating wavelet decomposition, the corresponding features were extracted at both levels of the two‐level decomposition, resulting in 224 features in total. These features were concatenated into a feature vector for each image, and the resulting feature vectors were aggregated by participant ID by averaging the corresponding feature values across all images contributed by the same participant, yielding one aggregated feature vector per participant for subsequent analyses. Statistical feature selection was performed exclusively on the training data to prevent information leakage from the validation and test sets.

Normality was assessed separately for each group using the Shapiro–Wilk test. Subsequently, appropriate statistical methods were selected based on the data distribution. When both groups of data satisfied the normal distribution, Levene's test for homogeneity of variances was further performed. If homogeneity of variances is satisfied, the independent samples *t*‐test was used; if not, the Welch‐corrected *t*‐test was employed. When at least one of the two groups does not satisfy the normal distribution, the Mann–Whitney *U* test was adopted. All tests were applied to participant‐level aggregated feature values rather than treating individual images as independent observations. All statistical tests were two‐sided, with the significance level set at *p* < 0.05. To account for multiple comparisons, the Benjamini–Hochberg procedure was applied to the raw *p*‐values of all between‐group comparisons between patients with diabetes and healthy controls included in the corresponding image representation to control the false discovery rate (FDR) at 0.05. Thus, 112 comparisons were corrected for representations without wavelet decomposition, whereas 224 comparisons were corrected jointly for the full WEMS‐TFE representation, covering both decomposition levels. Statistical significance was determined using the adjusted *p*‐values.

Although statistical significance testing can determine whether between‐group differences are statistically significant, its results are easily influenced by sample size and cannot directly reflect the actual magnitude of differences. Especially under small sample conditions, reliance solely on significance testing may be insufficient to fully characterize between‐group feature differences. Therefore, this study further introduced the effect size calculation method and threshold criteria proposed by Fritz et al.^31^ to quantitatively evaluate the degree of differences. Effect sizes were calculated using participant‐level aggregated feature values. The specific calculation formulas are as follows:

For cases where both groups satisfy the normal distribution, Hedges’ g is used as the effect size indicator for mean differences between two groups, quantifying the actual effect size of differences while correcting for small‐sample bias.${\bar{X}}_{1}$ and ${\bar{X}}_{2}$ are the means of the two groups, and $S_{1}$ and $S_{2}$ are the standard deviations of the two groups. The same pooled standard deviation formula was used regardless of whether the homogeneity of variances assumption was satisfied:

$$s_{p}=\sqrt{\frac{\left(n_{1}-1\right)s_{1}^{2}+\left(n_{2}-1\right)s_{2}^{2}}{n_{1}+n_{2}-2}}$$

and Hedges’ g is computed using the pooled standard deviation and a correction factor:

(7) $$g=J\left(\nu \right)\frac{{\bar{x}}_{1}-{\bar{x}}_{2}}{s_{p}},\;\;\;\;\nu =n_{1}+n_{2}-2$$

where the small‐sample correction factor is J(ν) defined as:

$$J\left(\nu \right)=\frac{\Gamma (\nu /2)}{\sqrt{\nu /2}\Gamma \left(\left(\nu -1\right)/2\right)}$$

Hedges’ g was calculated using the pooled standard deviation and the small‐sample correction factor for both the independent‐samples *t*‐test and Welch's *t*‐test, regardless of the variance‐homogeneity result. The signed point estimate was calculated as shown in Equation (7), with positive values indicating higher mean feature values in patients with diabetes than in healthy controls. The absolute value of Hedges’ g was used for effect‐size thresholding. The following effect size thresholds are applied: 0.2 (small), 0.5 (medium), and 0.8 (large).

For cases where at least one group does not satisfy the normal distribution, Cohen's d and Hedges’ g are not appropriate. Therefore, the effect size r was calculated using the standardized effect‐size formulation associated with the Mann–Whitney *U* test, where z was obtained by transforming the two‐sided asymptotic *p*‐value of the Mann–Whitney *U* test, and N is the total sample size of the two groups.

(8) $$r=\frac{sign({\bar{x}}_{1}-{\bar{x}}_{2})|Z|}{\sqrt{N}}$$

The absolute standardized value was obtained as

$$\left|Z\right|=\Phi^{-1}\left(1-\frac{p_{\mathrm{MW}}}{2}\right)$$

where $p_{\mathrm{MW}}$ denotes the two‐sided asymptotic p‐value and $\Phi^{-1}$ is the inverse cumulative distribution function of the standard normal distribution. The sign of r was determined by the difference between the group means, rather than by the sign of the U statistic. Positive effect‐size values indicate higher mean feature values in patients with diabetes than in healthy controls, whereas negative values indicate the opposite direction.

The magnitude of the effect size was interpreted using the thresholds of 0.1 (small), 0.3 (medium), and 0.5 (large).

Based on the effect size calculation, this study further applied a large‐effect threshold as the final inclusion criterion. Specifically, for cases where Hedges’ g was used, only indicators with |g| ≥ 0.8 were retained; for cases where the *U*‐test correlation coefficient was used, only indicators with |r| ≥ 0.5 were retained. Only indicators meeting the large‐effect threshold were included in subsequent analyses.

All statistical analyses were implemented in Python 3.11.8, primarily using the scipy.stats module from SciPy 1.14.1 for statistical testing.

### 2.8 Performance evaluation of extracted features

Relying solely on statistical difference analysis cannot fully capture the effectiveness of feature combinations in empirical clinical tasks. As demonstrated by Latif et al.,^32^ after feature extraction and denoising of medical images, validating feature effectiveness using machine learning classifiers is an indispensable step.

Given that different models operate through distinct feature modeling mechanisms, employing multiple classes of models—including tree‐based, linear, distance‐based, and kernel‐based approaches—provides a more comprehensive assessment of feature expressiveness and reduces the dependence of the evaluation on any single modeling framework. Decoux et al. ^33^ demonstrated that classifier selection is an important factor influencing radiomics model performance, while no universally optimal classifier was identified across datasets. This finding supports the use of a limited but diverse set of classifiers to evaluate the discriminative information captured by the extracted features under different modeling mechanisms. We therefore adopted seven commonly used machine learning models: logistic regression (LR ^34^), decision tree (DT ^35^), random forest (RF ^36^), k‐nearest neighbor (KNN ^37^), support vector machine (SVM ^38^), XGBoost,^39^ and LightGBM.^40^ This selection enables a systematic evaluation of the extracted feature combinations across different classification mechanisms. Comparing their performance across feature extraction schemes also helps assess whether the discriminative information is consistently captured across classifiers.

To prevent information leakage between data subsets, participants were used as the unit of data partitioning, whereas image‐derived feature vectors were used as the unit of model input and performance calculation. The participant‐disjoint held‐out set was randomly selected at a ratio of 15% using a fixed random seed of 42 and comprised 18 participants (7 healthy controls and 11 patients with diabetes), contributing 45 image‐derived feature vectors (23 from healthy controls and 22 from patients with diabetes). The model‐development set comprised the remaining 105 participants (40 healthy controls and 65 patients with diabetes), corresponding to the remaining 85% of participants and contributing 244 image‐derived feature vectors (110 from healthy controls and 134 from patients with diabetes). Each feature vector was derived from a single ultrasound image and multiple feature vectors could originate from the same participant. All feature vectors from a given participant were assigned exclusively to the same data subset. The development set underwent five‐fold cross‐validation repeated five times. Each repetition used a different random seed (41–45) to generate a new five‐fold partition of the participants, yielding 25 fold‐level evaluations per classifier. Within each fold, participants were divided into training, validation, and test subsets at a ratio of 0.6:0.2:0.2, with all feature vectors from a given participant kept in the same subset. Model performance was calculated over the image‐derived feature vectors in the test subset. On average, each fold contained approximately 63 participants in the training subset, 21 participants in the validation subset, and 21 participants in the test subset. At the image‐derived feature‐vector level, this corresponded to approximately 146, 49, and 49 vectors per fold. For models not requiring a separate validation set, the training and validation subsets were combined for model fitting.

For each feature extraction scheme, feature selection was performed independently within each fold before machine learning model training. Specifically, the statistical feature screening procedure described in Section 2.7 was first performed using the training subset. The statistically filtered feature vectors could still contain a substantial number of features, potentially increasing the risk of overfitting. Therefore, further feature reduction was performed using Pearson correlation analysis followed by L1‐regularized logistic regression‐based feature selection. Pearson correlation analysis was used to remove highly redundant features, with one feature removed from pairs showing an absolute correlation coefficient greater than 0.90. The remaining features were then standardized using the training subset and subjected to L1‐regularized logistic regression, with the regularization parameter optimized by three‐fold StratifiedGroupKFold cross‐validation grouped by participant ID, over 50 logarithmically spaced C values from 10^−^^4^ to 10^1^ using ROC‐AUC (Area Under the Receiver Operating Characteristic Curve) as the selection criterion. All image‐derived feature vectors from the same participant were kept within the same inner fold. Features with non‐zero coefficients were retained, with a minimum of eight features imposed when necessary. The resulting feature set was then applied unchanged to the validation and test subsets, and the selected features were used for model training and evaluation.

Seven machine learning classifiers were evaluated using the same participant‐level data partitions. For classifiers requiring hyperparameter optimization, hyperparameters were optimized using either randomized search or grid search with three‐fold StratifiedGroupKFold cross‐validation, with participant IDs supplied as grouping variables and ROC‐AUC as the optimization criterion. DT was evaluated using a fixed parameter configuration without hyperparameter search. For XGBoost and LightGBM, fixed hyperparameter configurations were used, with validation‐based early stopping applied where applicable.

For models requiring hyperparameter optimization, the search spaces included regularization strength, class weighting, model complexity, and neighborhood or ensemble‐related parameters, as appropriate to each classifier. LR and SVM used randomized search over C∈{0.01,0.02,0.05,0.1,0.5,1.0} and C∈{0.001,0.005,0.01,0.05,0.1,0.5}, respectively, together with class‐weighting options (“balanced” or None), while RF additionally considered the number of trees (50 or 100), tree depth (3 or 4), and minimum leaf size (4 or 8). The randomized searches for LR, SVM, and RF used 10, 12, and 10 iterations, respectively, with random_state = 42. KNN used grid search for the number of neighbors (3, 5, 7, 9, 11, or 15) and weighting strategy (“uniform” or “distance”), using the Minkowski distance with *p* = 2 (Euclidean distance). DT was evaluated using a fixed configuration (max_depth = 2, min_samples_leaf = 8), without hyperparameter search. LR used an L1 penalty with the liblinear solver, whereas SVM used a linear kernel; accordingly, the gamma parameter was not applicable. RF used the Gini impurity criterion and max_features = ‘sqrt’. XGBoost and LightGBM used predefined configurations with a learning rate of 0.01, L1 and L2 regularization coefficients of 1.0 and 5.0, respectively, and a maximum tree depth of 2. XGBoost used 200 maximum boosting rounds, subsampling ratios of 0.8 for both rows and columns, and log‐loss‐based early stopping with a patience of 20 rounds. LightGBM used GBDT boosting with two leaves per tree and a maximum of 50 boosting rounds, with binary log‐loss‐based early stopping using the same patience. Both models used a random seed of 42. In this study, patients with diabetes were defined as the positive class, and healthy controls were defined as the negative class. Classification performance was assessed using F1‐score and ROC‐AUC as the primary indicators of classification performance and discrimination ability, respectively. Results across the 25‐fold evaluations were summarized using the mean, standard deviation (SD), and 95% confidence interval (CI). All analyses were performed using Python 3.11.8 with scikit‐learn 1.5.2, XGBoost 3.0.5, and LightGBM 4.6.0.

### 2.9 Design of ablation study on feature extraction schemes

The proposed WEMS‐TFE method integrates multi‐scale wavelet decomposition with GLCM‐based texture feature extraction. To evaluate the contributions of its design components, five feature extraction schemes (S1–S5) were designed, as summarized in Table 1. The ablation study examined three aspects: the contribution of wavelet‐based feature extraction, the effect of decomposition levels, and the choice of wavelet basis function.

**TABLE 1:** Definition of the evaluated feature extraction schemes.

Scheme	Method Name	Description
S1	Ours (WEMS‐TFE)	Proposed WEMS‐TFE combining multi‐scale wavelet transform and GLCM
S2	Baseline	Baseline: GLCM‐based feature extraction without wavelet transform
S3	Ours w/ Level‐1 Only	Ablation: using only level‐1 wavelet coefficients with GLCM
S4	Ours w/ Level‐2 Only	Ablation: using only level‐2 wavelet coefficients with GLCM
S5	Ours w/ sym4	Ablation: replaced Haar with sym4 as the wavelet basis

#### 2.9.1 Contribution of wavelet‐based feature extraction

Conventional GLCM features are extracted directly from the original image without wavelet decomposition. To investigate the contribution of wavelet‐based feature extraction, S2 applies GLCM directly to the original image, whereas S1 applies GLCM to multi‐scale Haar wavelet coefficients. The comparison between S1 and S2 evaluates the overall effect of incorporating wavelet‐based decomposition into the feature extraction scheme.

#### 2.9.2 Effect of decomposition levels

Image textures contain information at different spatial scales, which may be captured by different wavelet decomposition levels. To investigate the contribution of each level, S3 and S4 used only the level‐1 and level‐2 Haar wavelet coefficients, respectively, while retaining the same GLCM‐based feature extraction procedure. Comparing S1 with S3 and S4 evaluated the effect of combining information from multiple decomposition levels. Since S2–S4 contain half as many features as S1, these comparisons reflect the combined effects of feature representation and dimensionality.

#### 2.9.3 Choice of wavelet basis function

The wavelet basis influences the decomposition filters and the resulting coefficients, thereby affecting the extracted texture features. Haar is a computationally simple, compactly supported wavelet with a discontinuous basis function, making it suitable for representing abrupt intensity changes and local details. In contrast, sym4 has greater smoothness and approximate symmetry, potentially providing a different representation of local texture patterns. To examine the sensitivity of the proposed method to the wavelet basis, S5 replaced Haar with sym4 while keeping all other settings identical to S1. Since S1 and S5 have the same feature dimensionality, their comparison provided a more direct assessment of the effect of wavelet basis selection.

Overall, the comparisons between S1 and S2–S5 evaluated the effects of wavelet‐based feature extraction, decomposition‐level selection, and wavelet basis selection under the specified experimental settings. All five schemes were evaluated using the same participant‐level data partitions, feature selection procedures, and seven machine learning classifiers described in Section 2.8.

### 2.10 Final feature extraction and model development strategy

Following completion of the ablation experiments, the five feature extraction schemes (S1–S5) were compared based on their overall performance and robustness across the seven machine learning classifiers. Overall performance was quantified using a composite score that equally weighted the mean F1‐score and mean ROC‐AUC for each scheme–classifier combination:

$$\mathrm{Composit}e_{s,m}=\frac{{\bar{F1}}_{s,m}+{\bar{\mathrm{ROC}0\mathrm{AUC}}}_{s,m}}{2},$$

where s denotes the feature extraction scheme and m denotes the classifier. The composite scores were then averaged across the seven classifiers to obtain the overall performance score for each feature extraction scheme:

$$P_{s}=\frac{1}{7}\sum_{m=1}^{7}\mathrm{Composit}e_{s,m}.$$

Robustness was quantified based on the variability of the composite score across the 25 fold‐level evaluations (five repeats × five folds). For each scheme–classifier combination, the standard deviation of the composite score across the 25 evaluations was calculated, and these seven per‐model SD values were averaged across the seven classifiers. The robustness score was then defined as the negative of this mean SD:

$$R_{s}=-\frac{1}{7}\sum_{m=1}^{7}SD_{25}\left(\mathrm{Composit}e_{s,m}\right).$$

Higher $P_{s}$ values indicate better overall cross‐model performance, whereas higher $R_{s}$ values (i.e., values closer to zero) indicate lower performance variability and greater robustness. The final feature extraction scheme was selected by jointly considering the overall performance and robustness scores, thereby accounting for both discriminative performance and stability across the seven classifiers and 25 fold‐level evaluations.

The selected feature extraction scheme was then applied to the entire 85% model development set. Unlike the repeated cross‐validation experiments described in Section 2.8, no separate validation set was used at this stage. Feature selection was performed anew using the complete development set, following the same sequential procedure applied within each cross‐validation fold: Statistical feature screening described in Section 2.7, Pearson correlation analysis to remove highly redundant features, and L1‐regularized logistic regression‐based feature selection. The resulting feature set was subsequently used to train each of the seven machine learning classifiers on the entire development set. No feature‐selection results obtained during the ablation experiments were reused.

The trained classifiers were then evaluated once on the participant‐disjoint held‐out set, which comprised 45 image‐derived feature vectors from 18 participants and had remained completely isolated from feature extraction scheme selection, feature selection, and model development throughout the study. Final model performance was assessed over these 45 image‐derived feature vectors using accuracy, precision, recall, specificity, F1‐score, ROC‐AUC, and PR‐AUC (Area Under the Precision‐Recall Curve). A confusion matrix was also computed at the image‐derived feature‐vector level. ROC and PR curves were generated at the image‐derived feature‐vector level to characterize the classification performance on the held‐out set.

To further investigate the consistency of the extracted discriminative information across different modeling mechanisms, feature importance was subsequently analyzed for all seven classifiers using the final feature set. Features showing consistent importance across multiple classifiers were identified for subsequent interpretation. A representative classifier was then selected according to predefined criteria for detailed SHAP‐based explainability analysis.

### 2.11 Artificial intelligence(AI) usage declaration

During the preparation of this manuscript, GPT‐5.6 Luna (OpenAI) and DeepSeek‐V4‐Pro (DeepSeek) were used to assist with English language polishing, grammar checking, and improvement of clarity and readability. The authors independently conducted the study design, methodology development, data analysis, interpretation of results, and scientific conclusions. All AI‐assisted suggestions and revisions were reviewed and verified by the authors, who take full responsibility for the accuracy, integrity, and content of the manuscript.

## 3 RESULTS

Manual ROI extraction demonstrated high inter‐observer agreement, with an overall mean IoU of 0.95 ± 0.03 across all pairwise comparisons. The mean IoU was 0.93 ± 0.04 between the two research investigators and 0.96 ± 0.03 between the investigators and the ultrasound physician.

### 3.1 Participant characteristics

Baseline demographic and clinical characteristics are summarized in Table 2. Patients with diabetes were significantly older than healthy controls (median age, 61 vs. 49 years; *P* < 0.001), and the sex distributions differed significantly between the two groups (*P* < 0.001). Height, weight, and body mass index (BMI) did not differ significantly between groups (all *P* > 0.05).

**TABLE 2:** Baseline demographic and clinical characteristics of healthy controls and patients with diabetes.

Clinical characteristics	Healthy controls (*n* = 47)	Patients with diabetes (*n* = 76)	*P* value
Age (years), median (IQR)	49(45–57)	61(55–68)	< 0.001
Sex (male, *n* [%])	7(14.9%)	52(68.4%)	< 0.001
Height (cm), mean ± SD	165.4 ± 6.4	167.5 ± 8.5	0.29
Weight (kg), mean ± SD	66.6 ± 7.4	71.4 ± 13.1	0.057
BMI (kg/m^2^), mean ± SD	24.4 ± 2.7	25.4 ± 3.9	0.247
Diabetes duration (years), median (IQR)	/	10(2–15)	/
HbA1c (%), median (IQR)	/	8.4(6.8–9.9)	/
Peripheral neuropathy (NDS ≥3), *n*/*N* (%)	/	38/76 (50.0%) overall; 38/65 (58.5%) among those with NDS assessments	/

*Note*: Data are presented as median (IQR) for age, diabetes duration, and HbA1c; mean ± SD for height, weight, and BMI; and *n* (%) for categorical variables (sex and peripheral neuropathy). For peripheral neuropathy, data are presented as overall proportion (*n* = 76) and proportion among patients with available NDS assessments (*n* = 65). Between‐group comparisons were performed using the Mann–Whitney *U* test or Welch's *t*‐test for continuous variables, depending on the results of the Shapiro–Wilk normality tests. Categorical variables were compared using the Pearson chi‐square test or Fisher's exact test, as appropriate. NDS assessments were unavailable for 11 patients with diabetes; no missing values were present for the other variables listed in the table. / indicates not applicable or not measured.

Abbreviations: IQR, interquartile range; SD, standard deviation; BMI, body mass index; HbA1c, glycated hemoglobin; NDS, neuropathy disability score.

Among patients with diabetes, the median disease duration was 10 years (IQR, 2–15), and the median glycated hemoglobin (HbA1c) level was 8.4% (IQR, 6.8–9.9). Peripheral neuropathy (NDS ≥3) was identified in 38 of 76 patients with diabetes (50.0% overall); among the 65 patients with available NDS assessments, the proportion was 38 of 65 (58.5%). NDS assessments were unavailable for 11 of the 76 patients with diabetes. No missing values were present for the other demographic and clinical variables summarized in Table 2.

### 3.2 Segmentation performance and quality assessment

The 95% bootstrap CIs were estimated using participant‐level cluster bootstrap resampling, with participants as the resampling units. The resulting H‐SAM model achieved mean Dice coefficients of 0.82 (95% bootstrap CI, 0.73–0.89) for the microchambers and 0.89 (95% bootstrap CI, 0.87–0.92) for the macrochambers (overall mean, 0.85; 95% bootstrap CI, 0.80–0.90). Because pixel spacing was not recorded, HD95 (95% Hausdorff distance) is reported in pixels rather than millimeters. The mean HD95 was 20.4 pixels (95% bootstrap CI, 15.9–24.4) for the microchambers and 60.2 pixels (95% bootstrap CI, 43.8–73.6) for the macrochambers, with an overall mean of 40.3 pixels (95% bootstrap CI, 29.7–48.3). These results indicate generally satisfactory segmentation performance, although the accuracy of boundary delineation cannot be conclusively established given the small test set. The masks generated by H‐SAM showed good agreement with manual segmentations performed by experienced physicians. The segmentation quality was subsequently reviewed by clinical experts before further processing.

### 3.3 Robustness of texture features to simulated segmentation errors

While Section 3.2 demonstrated high overall segmentation accuracy, residual boundary discrepancies are inevitable in practice. To assess the robustness of the proposed WEMS‐TFE feature representation to segmentation uncertainty, the sensitivity analysis was performed using the complete WEMS‐TFE configuration (S1). This analysis specifically evaluated whether the final feature extraction scheme was stable under simulated segmentation perturbations before comparing alternative feature extraction schemes in the subsequent ablation study.

Feature stability decreased with increasing perturbation magnitude. Dilation was better tolerated than erosion under ± 2 px perturbations, whereas the opposite was observed at ± 5 px (Table 3). Importantly, the WEMS‐TFE features remained robust to minor segmentation errors: Under ± 2 px perturbations, median ICCs remained ≥ 0.90, supporting a ≤ 2 px segmentation tolerance in the WEMS‐TFE pipeline, while larger perturbations resulted in reduced stability and highlighted the need for contour quality control.

**TABLE 3:** Sensitivity of WEMS‐TFE features to simulated segmentation errors (erosion −2/−5 px; dilation +2/+5 px).

Perturbation	Median ICC	ICC IQR	ICC ≥ 0.9, *n* (%)	Median Change, %	Change IQR, %
Erosion ‐2px	0.902	(0.839–0.939)	116 (51.8%)	5.84	(2.18–10.64)
Dilation +2px	**0.986**	(0.846–0.999)	**158 (70.5%)**	**0.21**	(0.09–0.64)
Erosion ‐5px	0.882	(0.601–0.947)	94 (42.0%)	4.75	(1.95–9.61)
Dilation +5px	0.812	(0.411–0.926)	68 (30.4%)	4.68	(2.04–9.82)

*Note*: Percentages for ICC ≥ 0.9 were calculated with a total size of *n* = 224, representing the number of WEMS‐TFE features analyzed.

### 3.4 Ablation results and analysis

As shown in Table 4 and Figure 5, S1 achieved the highest overall performance (0.9475), closely followed by S2 (0.9470), S5 (0.9451), S4 (0.9395), and S3 (0.9027). S1 also showed the highest robustness score (−0.0331), corresponding to the lowest average performance variability across the seven classifiers and 25 fold‐level evaluations. Although S2 achieved a comparable overall performance (a difference of only 0.0005), its robustness score was lower (−0.0434), indicating greater variability across fold‐level evaluations. S5 and S4 showed intermediate overall performance and relatively high robustness, with robustness scores of −0.0337 and −0.0364, respectively. S3 had the lowest overall performance (0.9027) and the lowest robustness score (−0.0654). The complete F1‐score and ROC‐AUC results for all five feature extraction schemes across the seven classifiers are provided in Appendix B (Tables B1 and B2), with values reported as mean ± SD and 95% confidence intervals (CIs). The confidence intervals are interpreted descriptively because the fold‐level estimates from repeated cross‐validation are correlated.

**TABLE 4:** Overall performance and robustness of S1–S5 across seven classifiers.

Scheme	Overall Performance (Mean)	Robustness (‐SD)
S1	**0.9475**	**−0.0331**
S2	0.9470	−0.0434
S3	0.9027	−0.0654
S4	0.9395	−0.0364
S5	0.9451	−0.0337

**FIGURE 5:**
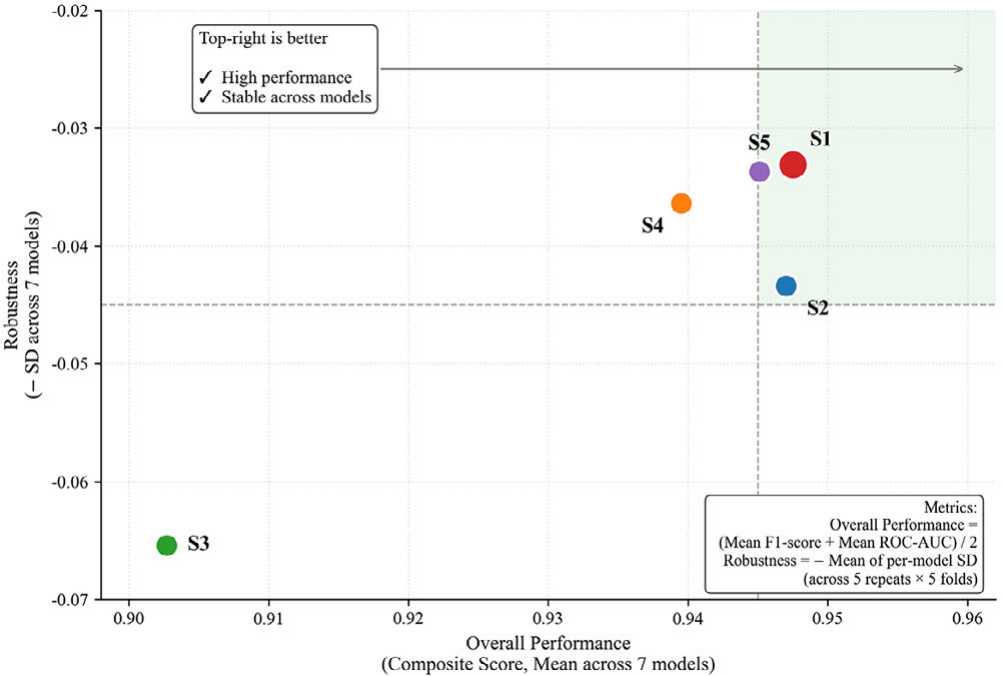
Overall performance versus robustness of S1–S5 across seven classifiers. Overall performance represents the mean composite score across the seven classifiers, where the composite score is the arithmetic mean of mean F1‐score and mean ROC‐AUC. Robustness is defined as the negative mean of per‐model SD across 25 fold‐level evaluations (5 repeats × 5 folds).

Taken together, S1 provided both the highest overall performance and the highest robustness among the five evaluated feature extraction schemes. Its overall performance (0.9475) was marginally higher than that of S2 (0.9470), while its higher robustness score (−0.0331 vs. −0.0434) indicated lower performance variability across repeated fold‐level evaluations. S1 also showed relatively stable performance across the seven classification models. Based on the predefined selection criteria described in Section 2.10, S1 was therefore selected as the final feature extraction scheme for subsequent analysis.

### 3.5 Final model performance and comparative analysis

Following the selection of S1 in the ablation analysis, the S1 feature extraction scheme was applied to the entire 85% model development set. Feature selection was subsequently performed anew using the complete development set, following the sequential procedure described in Section 2.10. The statistically significant features with large effect sizes retained at the preceding statistical filtering stage are summarized in Appendix C (Table C), including their signed effect sizes, 95% CIs, FDR‐adjusted *p*‐values, and corresponding effect size measures. Starting from these statistically filtered features, the feature dimensionality decreased from 47 features before Pearson correlation filtering to 19 features after Pearson filtering and further to 8 features after L1‐regularized logistic regression‐based feature selection. The resulting feature set was used to train the seven machine learning classifiers.

To provide a representative evaluation of the final classification model on the independent held‐out set, XGBoost was selected for detailed performance and explainability analysis. Although KNN yielded slightly higher numerical metrics under the S1 feature scheme (ROC‐AUC = 0.987, F1‐score = 0.973), XGBoost achieved strong predictive performance, with an ROC‐AUC of 0.977 and an F1‐score of 0.936 in the 25 fold‐level evaluations. The 95% CIs for the two classifiers overlapped substantially; however, this overlap alone does not establish statistical equivalence or the absence of a statistically significant difference. XGBoost also exhibited relatively low variability across fold‐level evaluations, with standard deviations of 0.022 for ROC‐AUC (the lowest among the seven classifiers) and 0.031 for F1‐score, and maintained ROC‐AUC values of at least 0.969 under four feature schemes (S1, S2, S4, and S5), suggesting consistent performance across these evaluated settings. Its tree‐based structure also provides a suitable basis for SHAP‐based interpretation, enabling the contribution of individual features to model predictions to be quantified and nonlinear feature effects to be examined. Unlike KNN, which does not provide native model‐based feature importance, XGBoost supports tree‐based importance measures and is compatible with SHAP (SHapley Additive exPlanations), enabling quantitative attribution of individual feature contributions to model predictions and the examination of feature interactions. These considerations motivated the selection of XGBoost as the representative classifier for the final independent held‐out evaluation and subsequent explainability analysis.

For the final model, XGBoost was trained using the 8 features retained after sequential Pearson correlation filtering and L1‐regularized logistic regression‐based feature selection. The final model retained the hyperparameter settings used during the cross‐validation stage, while the number of boosting estimators (n_estimators) was optimized using GridSearchCV over {50, 100, 200, 300}, with three‐fold StratifiedGroupKFold cross‐validation grouped by participant ID and ROC‐AUC as the selection criterion. Early stopping was not used during this final hyperparameter search, and the optimal number of boosting estimators was 100. A classification threshold of 0.5 was applied to the predicted probabilities. When evaluated on the 45 image‐derived feature vectors from the participant‐disjoint held‐out set, the representative XGBoost classifier achieved an accuracy of 0.9333, precision of 0.8800, recall (sensitivity) of 1.0000, specificity of 0.8696, F1‐score of 0.9362, ROC‐AUC of 0.9407, and PR‐AUC of 0.9283.

The corresponding confusion matrices are shown in Figure 6(a,b). The raw confusion matrix presents the numbers of correctly and incorrectly classified image‐derived feature vectors, whereas the normalized matrix shows the classification proportions within each true class. These matrices characterize the image‐derived feature‐vector‐level classification behavior of the representative XGBoost model on the participant‐disjoint held‐out set.

**FIGURE 6:**
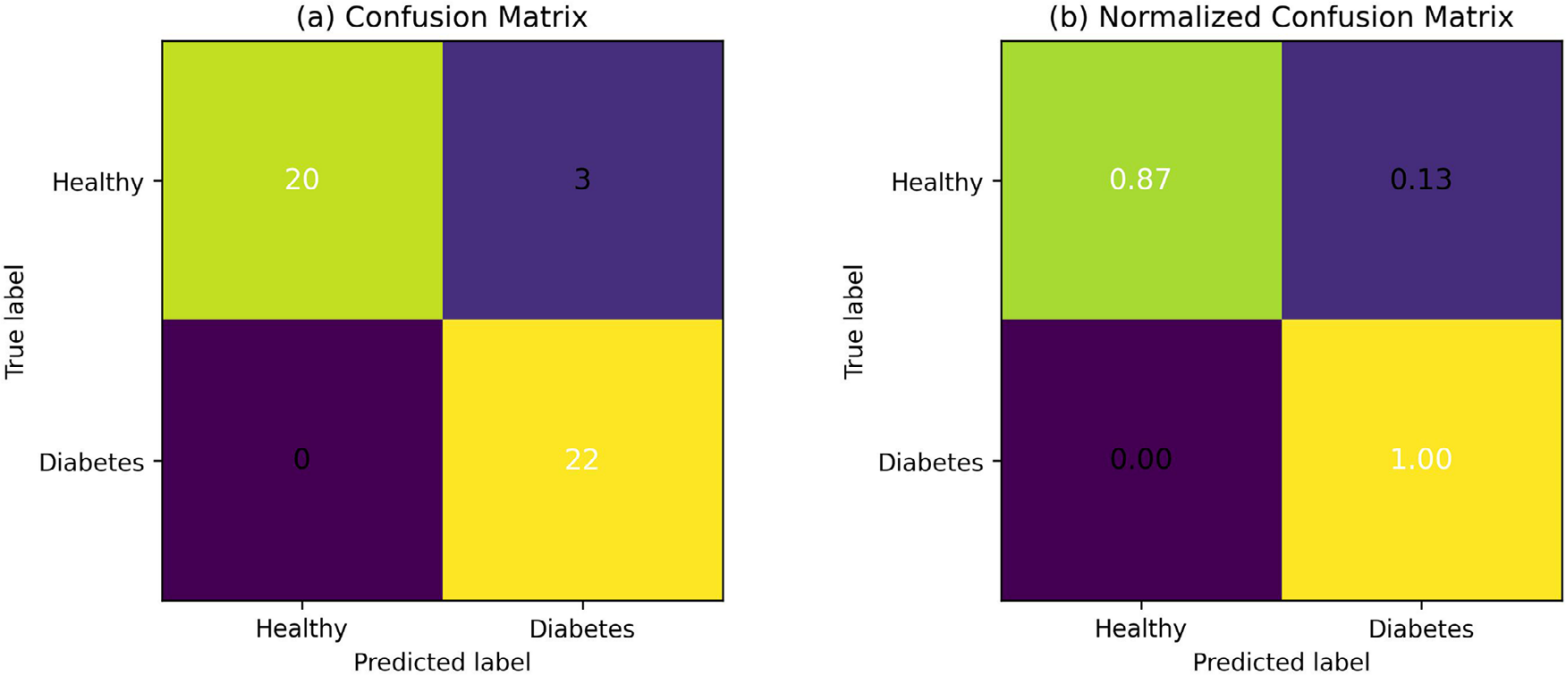
Confusion matrices of the representative XGBoost classifier on the participant‐disjoint held‐out set. (a) Raw confusion matrix showing the numbers of correctly and incorrectly classified image‐derived feature vectors. (b) Normalized matrix showing the corresponding proportions within each true class.

The ROC and PR curves are presented in Figure 7(a,b), providing complementary views of the discrimination and precision‐recall characteristics of the representative classifier at the image‐derived feature‐vector level on the participant‐disjoint held‐out set.

**FIGURE 7:**
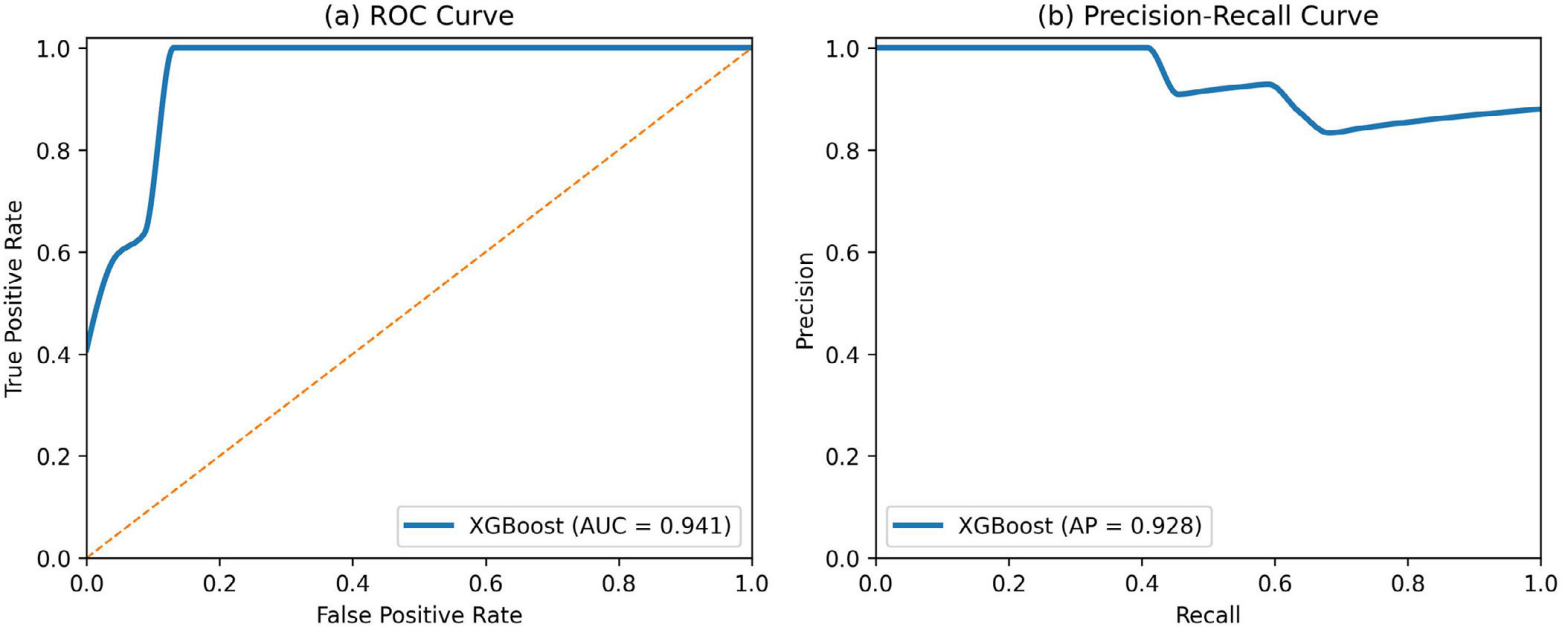
Receiver operating characteristic and precision‐recall curves of the representative XGBoost classifier on the participant‐disjoint held‐out set. (a) ROC curve. (b) Precision‐recall curve. The curves were generated using predictions for image‐derived feature vectors. For visualization, the empirical ROC and PR curves were rendered using shape‐preserving interpolation, whereas ROC‐AUC and PR‐AUC were calculated from the original prediction probabilities without interpolation.

### 3.6 Cross‐model feature importance and SHAP‐based explainability analysis

To examine whether the identified discriminative features were consistently recognized across different classification mechanisms, feature importance was independently assessed for all seven classifiers trained using the final 8‐feature S1 representation. The training procedures for all seven classifiers followed the final‐model training procedure described in Section 3.5. For classifiers with predefined search spaces, hyperparameter optimization was repeated on the model development set using the search spaces specified in Section 2.8, and the best‐performing configuration was selected for each classifier. As specified in Section 3.5, early stopping was disabled for XGBoost, and the same setting was applied to LightGBM.

Feature importance was defined as the absolute value of the standardized coefficient for LR, the absolute value of the standardized linear‐kernel weight for SVM, Gini impurity‐based importance for DT and RF, weight‐based importance for XGBoost, split‐based importance for LightGBM, and permutation importance (computed on the training set with 10 repetitions using ROC‐AUC) for KNN.

For each classifier, the five highest‐ranked features were identified, and the frequency with which each feature appeared in the top‐5 lists was subsequently calculated across the seven classifiers. Features appearing in at least three of the seven top‐5 lists were summarized in Table 5.

**TABLE 5:** Features recurrently ranked among the top 5 across the seven machine learning classifiers.

Feature	Top‐5 Frequency	Algorithms
L1_correlation_45°(l)	7/7	LR, DT, XGBoost, SVM, KNN, RF, LightGBM
L2_correlation_90°(l)	7/7	LR, DT, XGBoost, SVM, KNN, RF, LightGBM
L2_correlation_135°(s)	7/7	LR, DT, XGBoost, SVM, KNN, RF, LightGBM
L2_info_meas1_0°(s)	4/7	LR, DT, XGBoost, KNN
L2_max_corr_coef_90°(l)	3/7	LR, SVM, RF
L2_info_meas1_45°(l)	3/7	DT, XGBoost, KNN

As shown in Table 5, three features appeared in the top‐5 lists of all seven classifiers (7/7): L1_correlation_45°(l), L2_correlation_90°(l), and L2_correlation_135°(s). L2_info_meas1_0°(s) appeared in the top‐5 lists of four classifiers (4/7), while L2_max_corr_coef_90°(l) and L2_info_meas1_45°(l) each appeared in three classifiers' top‐5 lists (3/7). The recurrence of these features across classifiers indicates that their importance was not restricted to a single classification mechanism.

Notably, most of the features identified as consistently important across the seven classifiers were also present in the SHAP summary plot (Figure 8), although the two feature sets were not completely identical. This concordance suggests that the features retained from the S1 feature extraction scheme after sequential feature selection showed consistent importance across different classification mechanisms, while a substantial proportion of these features also contributed to the predictions of the representative XGBoost classifier.

**FIGURE 8:**
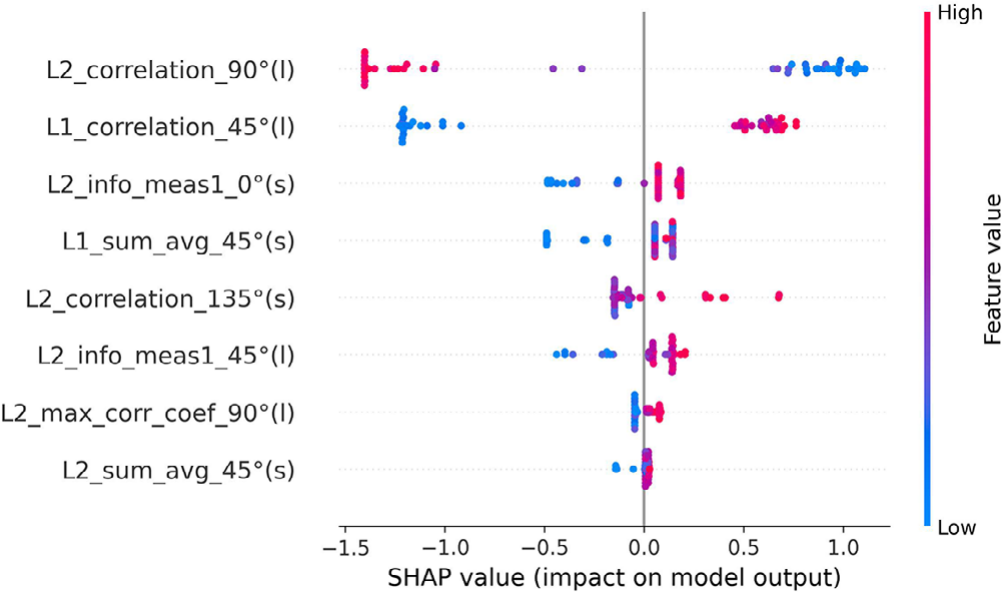
SHAP summary plot of the representative XGBoost classifier, showing feature contributions and their corresponding feature values.

The SHAP summary plot (Figure 8) further characterized the contribution patterns of the selected features within the XGBoost model. SHAP values were computed for the image‐derived feature vectors in the participant‐disjoint held‐out set using the TreeExplainer implementation in SHAP 0.48.0, which derives the expected value from the tree structure. Each point in the summary plot represents one image‐derived feature vector, with the horizontal position indicating the direction and magnitude of the feature contribution to the model output and the point color representing the corresponding feature value, from low to high. Here, the prefixes L1 and L2 denote features extracted from the level‐1 and level‐2 wavelet decomposition sub‐bands, respectively, while the suffixes (s) and (l) indicate features extracted from the microchamber and macrochamber regions, respectively. The features are ordered by their mean absolute SHAP values, with higher‐ranked features exhibiting greater overall contributions to the model output. L2_correlation_90°(l) showed the largest overall contribution to the model output, followed by L1_correlation_45°(l), L2_info_meas1_0°(s), and L1_sum_avg_45°(s). For L2_correlation_90°(l), lower feature values were predominantly associated with negative SHAP values, whereas higher values tended to contribute positively to the model output, indicating a pronounced value‐dependent contribution pattern. L1_correlation_45°(l) also showed a clear value‐dependent contribution pattern, with higher feature values mainly associated with positive SHAP values and lower values contributing negatively. L1_sum_avg_45°(s) and L2_info_meas1_0°(s) also showed a tendency toward positive SHAP contributions at higher feature values, although these patterns were less pronounced.

From an image‐texture perspective, the correlation features quantify the statistical relationship between neighboring gray levels in the wavelet‐derived representations. The recurrence of these correlation features across classifiers, together with the value‐dependent SHAP contribution patterns observed for selected correlation features in XGBoost, suggests that the corresponding gray‐level relationships contained discriminative information for distinguishing patients with diabetes from healthy controls.

Overall, the cross‐model feature‐importance analysis identified a set of features that recurred across multiple classification mechanisms, while the SHAP analysis further revealed their model‐specific contribution directions and magnitudes. The convergence of these analyses supports the consistency of the discriminative information captured by the final S1 feature representation and provides model‐specific insight into how selected features contributed to the XGBoost predictions.

## 4 DISCUSSION

### 4.1 Summary of key findings

This study established an explainable machine learning framework based on multi‐scale texture analysis for plantar ultrasound images. By comparing heel pad texture features between patients with diabetes and healthy controls, the framework identified group‐associated differences across multiple texture dimensions in both the microchamber and macrochamber regions. The WEMS‐TFE‐based S1 scheme achieved the highest predefined composite performance and robustness among the five evaluated feature extraction schemes across the seven classifiers, supporting the value of the multi‐scale feature representation under the present experimental design. These findings provide imaging‐based evidence of group‐level heel pad ultrasound texture differences, which may be affected by residual demographic confounding and should not be interpreted as evidence of diabetes‐specific structural mechanisms.

### 4.2 Comparison with prior work

Current investigations into the pathogenesis of DF lesions rely primarily on force plates, elastography, and ultrasonography to assess muscle function, soft‐tissue mechanical properties, and morphological changes.^4^, ^5^, ^6^ These studies have revealed structural and functional abnormalities in the plantar tissue of patients with diabetes, providing an important foundation for understanding diabetes‐associated plantar tissue changes.

Diabetes has been associated with metabolic, vascular, and connective‐tissue changes that may influence the structural and mechanical characteristics of plantar soft tissue. Such tissue‐level differences may be reflected in ultrasound echo distributions and texture patterns.

However, existing ultrasound‐based studies have notable limitations. Puri et al. used DWT‐derived texture energy features ^9^ with plantar stiffness measurements to distinguish the subcutaneous microstructure of patients with diabetes from that of healthy controls. Although this work demonstrated the potential of ultrasound texture analysis for characterizing diabetes‐associated tissue differences, its analysis was limited to frequency‐based texture energy features and did not further explore richer textural information. Duan et al. subsequently examined structural differences across distinct layers of the heel pad, applying selected GLCM texture features.^8^ Although statistically significant differences in the macrochamber were identified between patients with diabetes and healthy controls, the approach provided limited characterization of the nonlinear, multi‐scale information in ultrasound textures and lacked independent validation of feature effectiveness.

In contrast to these prior studies, the present work adopts a multi‐scale texture analysis strategy based on a two‐level 2D‐DWT decomposition, followed by multi‐directional GLCM feature extraction across four orientation directions. This design provides a broader representation of ultrasound texture information across spatial scales and orientations. The discriminative consistency and robustness of the resulting features were further evaluated through multiple machine learning models of different architectures. Importantly, the ablation results should not be interpreted as demonstrating uniform superiority of WEMS‐TFE over conventional single‐scale GLCM for every classifier or individual performance metric. Rather, under the predefined composite evaluation that equally weighted F1‐score and ROC‐AUC and incorporated performance variability across repeated cross‐validation evaluations, the S1 WEMS‐TFE scheme achieved the highest overall performance and robustness among the five evaluated schemes.

The seven classifiers were therefore used primarily as validation tools rather than as a separate methodological contribution. By including linear, tree‐based, distance‐based, and kernel‐based modeling mechanisms, the analysis examined whether the discriminative information captured by the feature representation remained informative across different classification strategies. This cross‐model consistency provides complementary evidence that the observed performance was not dependent on a single classifier architecture.

### 4.3 Feature interpretability and research implications

To address the interpretability limitations of existing ultrasound texture‐based approaches, this study integrated feature importance analysis with SHAP‐based interpretation to provide feature‐level information on the consistency and model‐specific contribution of discriminative texture features. Across the seven classifiers, 6 features were repeatedly identified as important, indicating that these features captured discriminative information that was relatively consistent across different modeling mechanisms. Most of these features also appeared among the important features in the XGBoost SHAP analysis, providing a high degree of concordance between the cross‐model importance analysis and the model‐specific explanation.

The two most representative features, L2_correlation_90°(l) and L1_correlation_45°(l), showed clear value‐dependent contribution patterns in the XGBoost SHAP analysis, with higher feature values generally associated with positive SHAP values and lower feature values predominantly associated with negative SHAP values. L1_sum_avg_45°(s) and L2_info_meas1_0°(s) also showed a tendency toward positive SHAP contributions at higher feature values, although these patterns were less pronounced. From an image‐texture perspective, these descriptors characterize neighboring gray‐level relationships and entropy‐related texture information within specific wavelet representations and orientations. These observations provide interpretable information about how selected texture descriptors contributed to the XGBoost predictions, but they should be regarded as image‐level associations rather than direct evidence of underlying tissue mechanisms or histopathological changes.

The involvement of both microchamber‐ and macrochamber‐derived features indicates that discriminative ultrasound texture information was present in more than one anatomical compartment of the heel pad. By linking quantitative texture descriptors with their potential image‐texture characteristics, the proposed interpretability framework provides additional insights into the features underlying model predictions. These findings should not be interpreted as evidence of a specific histological or biomechanical mechanism. Rather, they demonstrate that selected ultrasound texture descriptors contain discriminative information associated with the diabetes and healthy‐control groups. Further histological, biomechanical, and longitudinal studies are required to determine the biological significance and temporal relevance of these image‐level associations.

### 4.4 Limitations

Several limitations of this study should be acknowledged.

1. Sample size and data source. The sample size is relatively modest (123 participants, 289 ultrasound images), and data were collected from a single center. This may constrain the generalizability of the findings. Larger, multicenter studies are needed to confirm these results.

In addition, because multiple ultrasound images from some participants contributed image‐derived feature vectors, the final held‐out performance was evaluated at the image‐derived feature‐vector level rather than the participant level. Accordingly, these performance estimates characterize classification at the feature‐vector level and should not be directly interpreted as participant‐level diagnostic performance.

1. Acquisition, ROI extraction, and segmentation variability. Ultrasound image acquisition is operator‐dependent, and all images in this study were collected using a single device (Aplio i900) with participants in a fixed position, which may limit the reproducibility and generalizability of the extracted features across different scanning protocols, devices, and patient positioning. ROI extraction, although achieving a mean inter‐observer IoU of 0.95 ± 0.03, relied on manual delineation guided by ultrasound physicians and may introduce variability in large‐scale or multi‐center applications. The H‐SAM model used for microchamber and macrochamber segmentation achieved a mean Dice coefficient of 0.85; however, the segmentation quality may vary when applied to images acquired under different ultrasound settings or from populations with more heterogeneous plantar anatomy.
2. Demographic variables including sex, age, height, weight, and BMI were collected and compared between the diabetes and healthy control groups. Significant differences in age and sex distribution were observed between the groups, whereas BMI did not differ significantly. In addition, diabetes type was not systematically recorded, PAD status was unavailable, and NDS assessments were unavailable for 11 patients with diabetes. These factors may contribute to the observed ultrasound texture differences. Because adjusted analyses were not performed, residual confounding cannot be excluded. Therefore, the observed findings should be interpreted as group‐associated ultrasound texture differences that may be affected by residual demographic confounding, rather than evidence of diabetes‐specific structural changes or causal tissue mechanisms.
3. Unilateral data acquisition. The right heel was selected for consistency with prior plantar soft tissue ultrasound protocols ^6^, ^7^, ^8^ and because right‐foot preference is the predominant phenotype in adults (non‐right‐footedness: 12.1%–23.7%).^41^ Foot preference was not individually verified, and the right heel was not assumed to be the dominant side; it was used as a standardized measurement site. Restriction of the analysis to the right heel may overlook bilateral asymmetry and microstructural changes elsewhere on the plantar surface.
4. Cross‐sectional design and clinical interpretation. The present study used a cross‐sectional comparison between patients with diabetes and healthy controls. Therefore, the observed texture differences cannot establish their temporal relationship with the development of DF complications or determine whether they precede future ulceration. The intended clinical relevance of the framework is therefore limited to characterizing group‐associated heel pad ultrasound texture differences in individuals without active foot ulcers, rather than diagnosing early DF disease or predicting future ulcer development. Prospective longitudinal studies are required to determine whether the identified texture characteristics remain stable over time and whether they are associated with subsequent clinical outcomes.

### 4.5 Future work

Several directions warrant further investigation. First, larger multicenter studies involving diverse populations and ultrasound acquisition protocols are needed to validate the robustness and generalizability of the proposed framework across different clinical settings. Second, future studies should investigate the impact of variations in ultrasound devices, scanning conditions, patient positioning, and automated segmentation strategies to improve the reproducibility of image‐derived features. Third, integrating comprehensive clinical assessments, particularly neuropathy severity and other diabetes‐related indicators, may help determine how ultrasound texture characteristics relate to clinical and functional measures. Prospective longitudinal studies may further clarify whether these texture characteristics are associated with subsequent development of DF complications. Fourth, extending data acquisition to bilateral feet and multiple plantar regions would provide a more complete understanding of the spatial distribution and heterogeneity of diabetes‐related tissue changes. Finally, establishing feature‐specific reference ranges based on larger and more diverse datasets may further improve the interpretability and potential clinical utility of ultrasound texture analysis.

## 5 CONCLUSION

This study collected heel pad ultrasound images from patients with diabetes without active foot ulcers and healthy controls. We developed an explainable machine learning framework incorporating the WEMS‐TFE module, and combined it with statistical feature selection, multi‐classifier validation, and SHAP interpretability analysis. The results showed that the WEMS‐TFE‐based S1 feature extraction scheme achieved the highest predefined composite performance and robustness among the five evaluated schemes across seven classifiers, supporting its ability to capture discriminative multi‐scale texture information under the present experimental design. Cross‐model feature‐importance analysis identified 6 repeatedly important features, most of which were also present among the important features in the XGBoost SHAP analysis. Two representative features showed clear value‐dependent contribution patterns, providing model‐specific insight into the texture information used for classification.

The proposed explainable framework offers an approach for quantifying group‐associated differences in heel pad ultrasound texture features in individuals without active foot ulcers. These findings provide imaging‐based evidence of differences between the diabetes and healthy‐control groups rather than evidence of diabetes‐specific tissue mechanisms. Because residual demographic confounding cannot be excluded, the results should be interpreted as exploratory findings of group‐associated differences and may support further longitudinal and multimodal investigations.

## AUTHOR CONTRIBUTIONS

Lei Yin, Liang Zhou and Tao Wu were responsible for project administration, review and editing. Xinlei Ji and Yiming Li collected and analyzed the data, and wrote the draft. Jiachen Guo and Yitian Zhang revised the manuscript and gave some advice. All authors reviewed the manuscript.

## Funding information

This work is funded by the Technical Standards Program Under the “ Shanghai Science and Technology Innovation Action Plan ” [Grant number 24DZ2201100]; The “Soft Science Research” Project under the “Shanghai High‐Level Institution Construction and Operation Plan” [Grant number 25692104500]; Shanghai University of Medicine & Health Sciences Key Researcher External Academic Mentorship Program [Grant number AMSCP‐24‐21‐02]; the National Natural Science Foundation of China [Grant number 62376152].

## CONFLICT OF INTEREST STATEMENT

The authors declare that they have no competing interests or conflicts of interest related to this work.

## ETHICAL APPROVAL AND INFORMED CONSENT

This study was conducted in accordance with the Declaration of Helsinki. Imaging data were collected from Huashan Hospital affiliated Fudan University, with all data strictly anonymized. This prospective study was approved by the Institutional Ethics Review Board of Huashan Hospital affiliated Fudan University (HIRB No. 2021‐01030). All participants provided informed consent and signed the relevant consent forms.

## Data Availability

The datasets used and analyzed during the current study are available from the corresponding author on reasonable request. The code used and analyzed during the current study are available from the corresponding author on reasonable request.

## APPENDIX A. TEXTURAL FEATURES BASED ON GLCM(HARALICK ET AL.)^28^

### A..1

[Table acm270888-tbl-0006]

**TABLE A acm270888-tbl-0006:** Definitions of the textural features used in this study.

Texture Features	Description
ASM(Angular Second Moment)	$f_{1}=\sum_{i}\sum_{j}{\{p\left(i,\;j\right)}^{2}$
Contrast	f2=∑n=0Ng−1n2{∑i=1Ng∑j=1|i−j|=nNgp(i,j)}
Correlation	$f_{3}=\frac{\sum_{i}\sum_{j}\left(i\;j\right)\;p\left(i,j\right)-\mu_{x}\mu_{y}}{\sigma_{x}\sigma_{y}}$ $\mu_{x}$, $\mu_{y}$, $\sigma_{x}$ and $\sigma_{y}$ are the means and standard deviations of $p_{x}$ and $p_{y}$.
Variance (Sum of Squares)	$f_{4}=\sum_{i}\sum_{j}{\left(i-\mu \right)}^{2}\;p\left(i,j\right)$
IDM(Inverse Difference Moment)	$f_{5}=\sum_{i}\sum_{j}\frac{1}{1+{\left(i-j\right)}^{2}}\;p\left(i,j\right)$
Sum Average	$f_{6}=\sum_{i=2}^{2N_{g}}ip_{x+y}\left(i\right)$
Sum Variance	$f_{7}=\sum_{i=2}^{2N_{g}}{\left(i-f_{8}\right)}^{2}\;p_{x+y}\left(i\right)$
Sum Entropy	$f_{8}=-\sum_{i=2}^{2N_{g}}p_{x+y}\left(i\right)\log\left\{p_{x+y}\left(i\right)\right\}$
Entropy	$f_{9}=-\sum_{i}\sum_{j}p\left(i,j\right)\log\left(p\left(i,j\right)\right)$
Difference Variance	$f_{10}=\mathrm{variance}\mathrm{of}p_{x-y}$
Difference Entropy	$f_{11}=-\sum_{i=0}^{N_{g}-1}p_{x-y}\left(i\right)\log\left\{p_{x-y}\left(i\right)\right\}$
Information Measures of Correlation	$f_{12}=\frac{HXY-HXY1}{\max\{HX,HY\}}$ $f_{13}={\left(1-\exp[-2.0(HXY2-HXY)]\right)}^{1/2}$ HX and HY are entropies of $p_{x}$ and $p_{y}$. $HXY=-\sum_{i}\sum_{j}p\left(i,j\right)\log\left(p\left(i,j\right)\right)$ $HXY1=-\sum_{i}\sum_{j}p\left(i,j\right)\log\left\{p_{x}\left(i\right)p_{y}\left(j\right)\right\}$ $HXY2=-\sum_{i}\sum_{j}p_{x}\left(i\right)p_{y}\left(j\right)\log\left\{p_{x}\left(i\right)p_{y}\left(j\right)\right\}$
Maximal Correlation Coefficient	$f_{14}={\left(\mathrm{Second}\mathrm{largest}\mathrm{eigenvalue}\mathrm{of}Q\right)}^{1/2}$ $Q\left(i,j\right)=\sum_{k}\frac{p(i,k)p(j,k)}{p_{x}\left(i\right)p_{y}\left(k\right)}$

p(i,j) is the normalized (i,j) ‐th element of the GLCM, obtained by dividing the original matrix element P(i,j) by the normalization constant R, and represents the co‐occurrence probability of gray levels i and j; $N_{g}$ denotes the number of quantized gray levels and determines the dimension of the GLCM; $p_{x}\left(i\right)$ denotes the marginal probability distribution obtained by summing p(i,j) over all values of j; $p_{y}\left(j\right)$ is similarly defined from the column marginal distribution, when the gray‐level distribution of the image is uniform, $p_{x}\left(i\right)\approx p_{y}\left(j\right)$; $p_{x+y}\left(k\right)$ is the sum of all p(i,j) satisfying $i+j=k(k=2,3,\text{…},2N_{g})$, describes the probability distribution that the sum of gray value i and adjacent gray value j is k; $p_{x-y}\left(k\right)$ is the sum of all p(i,j) satisfying $\left|i-j|=k(k=0,1,\text{…},N_{g}-1\right)$, describes the probability distribution that the absolute difference of gray value i and adjacent gray value j is k.

## APPENDIX B. DETAILED PER‐MODEL RESULTS

### B..1

**TABLE B1:** F1‐score of different schemes (S1–S5) across 7 models.

Scheme	LR	DT	XGBoost	SVM	KNN	RF	LightGBM
S1	0.939 ± 0.030 (0.927–0.951)	0.893 ± 0.041 (0.876–0.909)	0.936 ± 0.031 (0.923–0.949)	0.931 ± 0.048 (0.911–0.951)	0.973 ± 0.028 (0.962–0.985)	0.968 ± 0.019 (0.960–0.976)	0.896 ± 0.041 (0.879–0.912)
S2	0.953 ± 0.030 (0.941–0.966)	0.918 ± 0.046 (0.899–0.937)	0.929 ± 0.051 (0.908–0.950)	0.929 ± 0.077 (0.897–0.961)	0.942 ± 0.040 (0.926–0.959)	0.943 ± 0.038 (0.928–0.959)	0.893 ± 0.067 (0.865–0.921)
S3	0.869 ± 0.064 (0.842–0.895)	0.868 ± 0.060 (0.843–0.892)	0.892 ± 0.054 (0.870–0.914)	0.847 ± 0.102 (0.805–0.889)	0.902 ± 0.061 (0.877–0.927)	0.909 ± 0.063 (0.883–0.935)	0.872 ± 0.063 (0.846–0.898)
S4	0.938 ± 0.028 (0.926–0.949)	0.887 ± 0.033 (0.873–0.901)	0.923 ± 0.036 (0.909–0.938)	0.896 ± 0.091 (0.858–0.933)	0.959 ± 0.024 (0.949–0.968)	0.943 ± 0.033 (0.929–0.956)	0.901 ± 0.042 (0.884–0.919)
S5	0.929 ± 0.035 (0.914–0.944)	0.935 ± 0.031 (0.922–0.948)	0.930 ± 0.036 (0.915–0.945)	0.923 ± 0.041 (0.906–0.940)	0.935 ± 0.044 (0.917–0.954)	0.942 ± 0.044 (0.924–0.960)	0.874 ± 0.040 (0.858–0.891)

**TABLE B2:** ROC‐AUC of different schemes (S1–S5) across 7 models.

Scheme	LR	DT	XGBoost	SVM	KNN	RF	LightGBM
S1	0.972 ± 0.029 (0.960–0.985)	0.933 ± 0.036 (0.918–0.948)	0.977 ± 0.022 (0.968–0.986)	0.971 ± 0.032 (0.958–0.984)	0.987 ± 0.025 (0.976–0.997)	0.983 ± 0.026 (0.972–0.994)	0.906 ± 0.055 (0.884–0.929)
S2	0.979 ± 0.026 (0.968–0.989)	0.945 ± 0.052 (0.923–0.966)	0.969 ± 0.037 (0.954–0.984)	0.978 ± 0.026 (0.967–0.989)	0.975 ± 0.027 (0.964–0.986)	0.971 ± 0.029 (0.959–0.983)	0.934 ± 0.061 (0.908–0.959)
S3	0.927 ± 0.079 (0.894–0.959)	0.906 ± 0.082 (0.872–0.940)	0.935 ± 0.054 (0.913–0.957)	0.925 ± 0.077 (0.893–0.957)	0.960 ± 0.042 (0.943–0.977)	0.953 ± 0.053 (0.931–0.975)	0.872 ± 0.061 (0.847–0.897)
S4	0.969 ± 0.032 (0.956–0.982)	0.934 ± 0.039 (0.918–0.950)	0.974 ± 0.023 (0.965–0.984)	0.970 ± 0.030 (0.958–0.982)	0.984 ± 0.022 (0.975–0.993)	0.982 ± 0.022 (0.973–0.991)	0.892 ± 0.054 (0.869–0.914)
S5	0.968 ± 0.031 (0.955–0.981)	0.956 ± 0.028 (0.945–0.968)	0.976 ± 0.024 (0.966–0.986)	0.959 ± 0.034 (0.945–0.973)	0.980 ± 0.023 (0.970–0.989)	0.986 ± 0.019 (0.979–0.994)	0.938 ± 0.042 (0.921–0.955)

## APPENDIX C. STATISTICALLY SIGNIFICANT FEATURES WITH LARGE EFFECT SIZES, INCLUDING SIGNED EFFECT SIZES, 95% CIS, ADJUSTED *P*‐VALUES, AND THE CORRESPONDING EFFECT SIZE MEASURES

### C..1

**TABLE C:** Statistically significant features with large effect sizes.

		Microchamber			Macrochamber		
Feature	Angle (°)	$p_{adj}$	Measure	Signed ES [95% CI]	$p_{adj}$	Measure	Signed ES [95% CI]
L1_contrast	45	< 0.001	Hedges’ g	0.961 [0.5426, 1.4592]	**/**	**/**	**/**
L1_contrast	135	< 0.001	Hedges’ g	0.9204 [0.506, 1.4316]	**/**	**/**	**/**
L1_variance	45	< 0.001	Hedges’ g	1.156 [0.7367, 1.6863]	**/**	**/**	**/**
L1_variance	135	< 0.001	Hedges’ g	1.1325 [0.7042, 1.6485]	**/**	**/**	**/**
L1_sum_avg	45	< 0.001	Hedges’ g	−1.0586 [−1.5554, −0.6495]	**/**	**/**	**/**
L1_sum_avg	135	< 0.001	Hedges’ g	−1.0623 [−1.5395, −0.6531]	**/**	**/**	**/**
L1_sum_var	45	< 0.001	r	0.5718 [0.3965, 0.7227]	**/**	**/**	**/**
L1_sum_var	135	< 0.001	r	0.5653 [0.3912, 0.713]	**/**	**/**	**/**
L1_correlation	45	**/**	**/**	**/**	< 0.001	Hedges’ g	0.8294 [0.472, 1.2257]
L1_correlation	135	**/**	**/**	**/**	< 0.001	Hedges’ g	0.8709 [0.5075, 1.293]
L1_info_meas1	45	**/**	**/**	**/**	< 0.001	r	−0.6417 [‐0.7484, −0.5184]
L1_info_meas1	135	**/**	**/**	**/**	< 0.001	r	−0.6464 [−0.7543, −0.5209]
L1_info_meas2	45	**/**	**/**	**/**	< 0.001	r	0.678 [0.5744, 0.7699]
L1_info_meas2	135	**/**	**/**	**/**	< 0.001	r	0.6817 [0.5765, 0.7736]
L2_contrast	45	< 0.001	Hedges’ g	1.1491 [0.6852, 1.8016]	**/**	**/**	**/**
L2_contrast	135	< 0.001	Hedges’ g	1.0158 [0.5586, 1.6242]	**/**	**/**	**/**
L2_correlation	45	**/**	**/**	**/**	< 0.001	r	0.6854 [0.5373, 0.7572]
L2_correlation	90	**/**	**/**	**/**	< 0.001	r	0.5697 [0.4396, 0.6848]
L2_correlation	135	< 0.001	Hedges’ g	1.9514 [1.4502, 2.6494]	< 0.001	Hedges’ g	1.9202 [1.4729, 2.507]
L2_variance	45	< 0.001	Hedges’ g	1.249 [0.7672, 1.9236]	< 0.001	Hedges’ g	1.0447 [0.6993, 1.4551]
L2_variance	135	< 0.001	Hedges’ g	1.218 [0.741, 1.859]	< 0.001	Hedges’ g	1.0285 [0.6912, 1.438]
L2_sum_avg	45	< 0.001	r	−0.6062 [−0.7334, −0.4579]	**/**	**/**	**/**
L2_sum_avg	135	< 0.001	r	−0.6215 [−0.7512, −0.472]	**/**	**/**	**/**
L2_sum_var	45	< 0.001	Hedges’ g	1.3359 [0.8603, 1.9961]	< 0.001	r	0.5625 [0.4143, 0.6907]
L2_sum_var	135	< 0.001	r	0.546 [0.346, 0.7185]	< 0.001	r	0.5579 [0.4139, 0.6911]
L2_info_meas1	0	< 0.001	r	−0.7741 [−0.8335, −0.6936]	< 0.001	r	−0.8102 [−0.8478, −0.7593]
L2_info_meas1	45	< 0.001	r	−0.8494 [−0.8604, −0.8249]	< 0.001	r	−0.8123 [−0.8507, −0.7606]
L2_info_meas1	90	< 0.001	r	−0.6148 [−0.7398, −0.4655]	< 0.001	r	−0.6422 [−0.7412, −0.5335]
L2_info_meas1	135	< 0.001	r	−0.8352 [−0.848, −0.8105]	< 0.001	r	−0.8182 [−0.8516, −0.7716]
L2_info_meas2	0	< 0.001	r	0.7945 [0.7248, 0.8432]	< 0.001	r	0.8292 [0.7897, 0.8562]
L2_info_meas2	45	< 0.001	Hedges’ g	1.2542 [1.0234, 1.5821]	< 0.001	r	0.8111 [0.7526, 0.8533]
L2_info_meas2	90	< 0.001	r	0.6041 [0.4568, 0.7344]	< 0.001	r	0.5954 [0.4729, 0.6999]
L2_info_meas2	135	< 0.001	Hedges’ g	1.2815 [1.045, 1.611]	< 0.001	r	0.817 [0.7673, 0.8533]
L2_max_corr_ coef	90	**/**	**/**	**/**	< 0.001	r	−0.6337 [−0.7446, −0.5083]

$p_{adj}$: Benjamini‐Hochberg FDR‐adjusted p‐value.

Feature naming convention: The prefixes L1 and L2 indicate that the corresponding texture features were extracted from the level‐1 and level‐2 wavelet decomposition sub‐bands, respectively.

Measure: Hedges’ g (Equation 7) was used for normally distributed data, with the pooled standard deviation (pooled SD) and a small‐sample correction factor applied regardless of whether equal variances were assumed in the statistical test. The effect size r (Equation 8) was used when at least one group did not satisfy normality (Shapiro–Wilk test, *p* < 0.05). As described in Section 2.7, the absolute value of this point effect size was used for large‐effect thresholding and subsequent feature selection. In the table, the signed effect size is additionally reported in the “Signed ES” column solely to indicate the direction of the difference. (C)

Signed ES [95% CI]: The sign indicates the direction of the effect size. Patients with diabetes were defined as the experimental group and healthy controls as the control group. Positive values indicate a higher mean feature value in patients with diabetes than in healthy controls, whereas negative values indicate the opposite direction. The 95% CIs for both Hedges’ g and the nonparametric effect size r were estimated using the percentile bootstrap method with 5000 resamples. In each resample, participants were independently sampled with replacement within each diagnostic group, using participant‐level aggregated feature values and preserving the sample size of each group. The corresponding effect size was recalculated for each resample using the same formula as for the point estimate. For r, the two‐sided asymptotic p‐value from each Mann–Whitney *U* test was transformed to |Z| using the inverse standard normal distribution, and the sign of the bootstrap effect size was determined by the difference between the group means. The 2.5th and 97.5th percentiles of the resulting bootstrap effect‐size distribution were used as the lower and upper CI bounds. The signed effect size and its 95% CI are reported for display only; feature selection was based on the absolute value of the point effect size (|g | ≥ 0.8 or |r | ≥ 0.5), as described in Section 2.7. All CIs were computed on the same samples used for the point estimates, before the large‐effect threshold was applied.
